# Reported Outcomes in Published Systematic Reviews of Interdisciplinary Pain Treatment: Protocol for a Systematic Overview

**DOI:** 10.2196/17795

**Published:** 2020-05-22

**Authors:** Elena Dragioti, Huan-Ji Dong, Britt Larsson, Björn Gerdle

**Affiliations:** 1 Pain and Rehabilitation Centre Department of Health, Medicine and Caring Sciences Linköping University Linköping Sweden

**Keywords:** Interdisciplinary pain treatment, multidisciplinary rehabilitation, chronic pain, outcome domains

## Abstract

**Background:**

Interdisciplinary pain treatment (IPT) is a complex intervention; its outcomes are very diverse, as are the methodologies for handling those outcomes. This diversity may hamper evidence-based decision making. Presently, there is no gold standard recommendation of how to select reported outcomes in published systematic reviews and meta-analyses to explicitly demonstrate the effectiveness of IPT.

**Objective:**

In this systematic overview, we aim to evaluate the reported outcome domains and measurements across published systematic reviews and meta-analyses and to identify any methods, considerations, and discussion regarding the handling of the chosen outcome domains and measurements.

**Methods:**

This article describes the protocol for a systematic overview of the outcomes reported in published systematic reviews and meta-analyses of randomized control trials for the effectiveness of IPT versus any control. To this end, we searched the PubMed, Cochrane Library, and Epistemonikos databases from inception to December 2019. Two independent investigators screened the titles, the abstracts of the identified records, and the full texts of the potentially eligible systematic reviews and meta-analyses, performed data extraction according to predefined forms, and rated the quality of the included systematic reviews and meta-analyses. The quality of the included systematic reviews and meta-analyses will be rated with AMSTAR (A MeaSurement Tool to Assess systematic Reviews) 2. Data will be analyzed descriptively and stratified by AMSTAR 2.

**Results:**

We introduced the rationale and design of a systematic overview to summarize and map the chosen IPT outcome domains and the methods of handling these outcomes reported in published systematic reviews and meta-analyses. As of December 2019, we collected 5229 systematic reviews, of which 147 (2.81%) were examined in-depth for eligibility. Topline results are anticipated by September 2020.

**Conclusions:**

The results of this study will be published as soon as they are available. Our results will fill a gap in the related literature and will be used to inform the development of a set of recommendations that can be applied in systematic reviews and hopefully serve as a gold standard.

**International Registered Report Identifier (IRRID):**

PRR1-10.2196/17795

## Introduction

Interdisciplinary pain treatment (IPT) is considered to be an optimal treatment option for chronic pain because it acknowledges the various pain complexities experienced by patients [1,2]. While many terms have been used to describe IPT in the literature (ie, multidisciplinary, multiprofessional, multimodal, and interprofessional), the International Association for the Study of Pain (IASP) has clarified the terminology for the different multicomponent treatments by defining IPT as “a multimodal treatment provided by a multidisciplinary team collaborating in assessment and treatment using a shared biopsychosocial model and goals” [3]. This definition makes a clear distinction between “multimodal treatment” and “multidisciplinary treatment” with respect to the biopsychosocial perspective.

As a result, IPT is based on a biopsychosocial framework provided by a team of professionals with distinct backgrounds; it contains one physical component and at least one educational, psychological, social, or occupational component [1-5]. Given this definition, the components of IPT can be activated independently or interdependently [6], leading to composite effects supported by known and unknown mechanisms. Each such effect is assumed to be an additive sum of the effects of its components [7]. As a result, IPT is a complex treatment [6,8]. Unlike pharmacological treatment, IPT targets the whole person rather than only targeting biochemical processes; therefore, complex patient conditions are paired with complex treatments [9,10].

Complex treatments such as IPT should incorporate multiple outcomes measured at multiple levels as well as strategies for handling those multiple outcomes [10,11]. For example, one systematic review including 46 randomized controlled trials (RCTs) reported a median of 9 outcomes per RCT [2]. However, outcomes in published systematic reviews are not usually divided into primary and secondary outcomes [4,5]. Additionally, the current practice for reporting RCTs is to analyze the outcomes as independent from one another [2,4,5]; meanwhile, a study from the Swedish Quality Registry for Pain Rehabilitation found significant intercorrelations between outcomes of RCTs [12]. Hence, the changes in these outcomes cannot be considered to be independent of each other because IPT is a complex treatment. This may mean that some outcomes are moderating and mediating variables; also, a change process occurs over time, with some changes occurring quickly while others occur more slowly.

Taken together, the great variation of the selected outcomes and procedures for handling multiple outcomes [2,13] may hamper direct and prompt comparison across RCTs in this field [14,15] and, thus, may hamper evidence interpretation [16]. Therefore, core outcome sets have been developed to standardize and improve the choice and reporting of outcome domains and to facilitate evidence-based decision making; examples include VAPAIN (Validation and Application of a core set of patient-relevant outcome domains to assess the effectiveness of multimodal PAIN therapy), IMMPACT (Initiative on Methods, Measurement, and Pain Assessment in Clinical Trials), and PROMIS (Patient-Reported Outcomes Measurement Information System) [16-18]. Despite these efforts, methods of reporting and handling the selected outcome IPT domains and measurements across the published systematic reviews and meta-analyses remain mostly unstudied. For example, in 2008, Scascighini et al [1] proposed an approach based on predefined primary and secondary outcomes and what is necessary to classify an intervention as positive before reviewing RCTs. However, other definitions of positive outcomes of an IPT already exist (eg, the majority of outcomes must be significantly better than for the control intervention) [4,5]. On the other hand, the Grading of Recommendations Assessment, Development and Evaluation (GRADE) approach used for evidence ratings in systematic reviews may not adequately describe the evidence base of complex treatments [19].

Given this background, the aim of this systematic review is to provide an overview of the IPT outcomes reported in systematic reviews and meta-analyses. More specifically, the objectives of this study are to evaluate the reported outcomes according to VAPAIN statements and IMMPACT and PROMIS recommendations [16-18] and to describe the methods, considerations, and discussion for handling the chosen outcome domains and measurements.

## Methods

This study protocol follows the Preferred Reporting Items for Systematic Review and Meta-Analysis Protocols (PRISMA-P) recommendations [20].

### Search Strategy

We searched the PubMed, Cochrane Library, and Epistemonikos databases from inception to December 31, 2019. A specific search strategy was developed for each database using the PubMed Systematic Reviews filter for systematic reviews and meta-analyses (see Textbox 1) combining MeSH keywords and other relevant terms, including multidisciplinary, interdisciplinary, patient care team, multidisciplinary biopsychosocial rehabilitation, chronic pain, and persistent pain, exploded when necessary.

**Database search strategy based on the PubMed Systematic Reviews filter.**

(“Chronic Pain”[Mesh] OR “Neuropathic Pain” [Mesh] OR chronic persistent pain [TIAB] AND ”Pain/rehabilitation”[Mesh] OR (”Pain/therapy”[Mesh] OR multidisciplinary [TIAB] OR interdisciplinary[TIAB] OR multimodal[TIAB]) OR multidisciplinary biopsychosocial rehabilitation [TIAB] AND ”Combined Modality Therapy” [Mesh] AND “Patient Care Team”[Mesh] NOT (”Neoplasms”[Mesh] OR surgery[TW]) AND (((systematic review[ti] OR systematic literature review[ti] OR systematic scoping review[ti] OR systematic narrative review[ti] OR systematic qualitative review[ti] OR systematic evidence review[ti] OR systematic quantitative review[ti] OR systematic meta-review[ti] OR systematic critical review[ti] OR systematic mixed studies review[ti] OR systematic mapping review[ti] OR systematic cochrane review[ti] OR systematic search and review[ti] OR systematic integrative review[ti]) NOT comment[pt] NOT (protocol[ti] OR protocols[ti])) NOT MEDLINE [subset]) OR (Cochrane Database Syst Rev[ta] AND review[pt]) OR systematic review[pt]

### Study Selection and Eligibility Criteria

We will include only systematic reviews (with and without meta-analyses) of RCTs investigating the effectiveness of IPT for any chronic pain condition as strictly defined by the original authors in the systematic review inclusion criteria (ie, pain lasting at least 3 months).

Meta-analyses that examined IPTs versus any control (eg, treatment as usual, waiting list) or other treatment (eg, physiotherapy, surgery) will be eligible for inclusion. If a systematic review examines various forms of therapies, it will be considered eligible only if separate results or analyses of IPT are presented.

The following inclusion criteria will be applied:

- To identify adequate systematic reviews, an IPT definition must be described in the full text and the involved IPT professionals should be clearly reported by the original authors.
- Only systematic reviews of RCTs published in peer-reviewed journals in English or Swedish will be included.
- At least 75% of participants will be people ≥18 years of age.
- At least 75% of participants will have chronic/persistent nociceptive and/or nociplastic pain (ie, for at least 3 months or more), such as chronic low back pain, chronic neck pain including whiplash-associated disorders, chronic widespread pain, fibromyalgia, chronic migraine and other headaches, myofascial pain syndromes, Ehlers-Danlos syndrome, hypermobility syndrome, and chronic neuropathic pain, such as painful diabetic neuropathy, trigeminal neuralgia, postherpetic neuralgia or spinal cord injury, multiple sclerosis, or stroke-related neuropathy.

Two independent investigators will screen the titles, the abstracts of the identified records, and the full texts of the potentially eligible articles. In cases of discrepancy, a third investigator will be consulted until agreement is reached.

We will exclude systematic reviews if they (1) review other meta-analyses (eg, meta-reviews, umbrella reviews), (2) include study designs other than RCTs, (3) include fewer than 75% of participants diagnosed with chronic pain, or (4) include a diagnosis of chronic pain due to cancer, infection, inflammatory arthropathy, osteoporosis, fracture, pregnancy, rheumatoid arthritis, or other rheumatic pain (eg, lupus, ankylosing spondylitis, psoriatic arthritis, Sjogren syndrome, polymyalgia rheumatica).

### Methodological Quality Assessment of Included Studies

Two independent investigators will rate the methodological quality of the selected systematic reviews using the AMSTAR 2 checklist [21]. The AMSTAR 2 is a 16-item instrument related to essential features of methodological rigor across systematic reviews. AMSTAR 2 does not generate an overall “score” but instead provides a rating scheme for the overall confidence in the results of the reviews as follows: high quality, moderate quality, low quality, or critically low quality [21].

### Data Extraction

Two independent investigators will abstract the data using predefined forms. For each eligible systematic review, we will record the Cochrane or PubMed ID, first author, publication year, chronic pain conditions, control/comparison arms, number of RCTs of IPTs included in the systematic review, outcomes investigated (primary and secondary if such categorization exists), outcome measurements, and total number of participants. Furthermore, we will extract data regarding the duration of the treatment (weeks and hours), treatment components, setting, and follow-up length. We will also record any method, strategy, considerations, or discussion regarding how the authors chose which outcomes to study and which methods to use to evaluate the evidence (eg, the GRADE approach).

### Data Synthesis

We will analyze data descriptively stratified by the methodological quality of the selected systematic reviews. We will provide the number of outcomes reported in each systematic review, the diversity of the reported outcomes, and the methodologies for outcome assessment. We will also evaluate the reported outcomes according to the VAPAIN statement on core pain outcome domains for IPTs [16], IMMPACT recommendations [17], and PROMIS recommendations [18]. According to VAPAIN, 8 core domains should be assessed in RCTs for IPT: pain intensity, pain frequency, physical activity, emotional well-being, satisfaction with social roles and activities, productivity (paid and unpaid, at home and at work, inclusive presentism and absenteeism), health-related quality of life, and the patient's perception of treatment goal achievement [16]. According to IMMPACT recommendations, the chronic pain trials should assess outcomes representing 6 core domains: pain, physical functioning, emotional functioning, participant ratings of improvement and satisfaction with treatment, symptoms and adverse events, and participant disposition (eg, adherence to the treatment regimen and reasons for premature withdrawal from the trial) [17]. Finally, according to PROMIS, the reported outcome domains should be classified in the following 3 core health areas: physical health (including the core health outcome domains of symptoms and function), mental health (including the core health outcome domains of affect, behavior, and cognition) and social health (including the core health outcome domains of relationships and function) [18]. We will also map and pinpoint any specific strategy by which the authors decided on the selected outcomes included in their systematic reviews and note whether there is any discussion on how to best evaluate the evidence of IPT, considering its treatment nature and complexity.

## Results

We have introduced the rationale and design of a systematic overview to summarize and map the chosen IPT outcome domains and the methods of handling these outcomes reported in published systematic reviews with meta-analyses. As of December 2019, we collected 5229 systematic reviews, of which 147 (2.81%) were examined in-depth for eligibility. Topline results are anticipated by September 2020.

## Discussion

The results of this systematic overview will fill a gap in the related literature and will be helpful to potential and practicing developers of IPT. By evaluating and mapping how the outcomes were selected and reported as well as which methods were used to evaluate the evidence in the published literature, we also hope to provide a proper way of framing the selection of research outcomes, which in turn may be a vital starting point to facilitate evidence synthesis and assessment of complex treatments for chronic pain in everyday clinical practice. The review results will be used to inform the development of a set of recommendations that can be applied in systematic reviews and hopefully serve as a gold standard.

Given the economic cost not only of pain itself but of its treatment, we expect that the results of this study will be of considerable interest to clinicians, academics, guideline developers, and policymakers; we will disseminate the findings widely through academic publications, conference presentations, and communication with health care providers.

## Abbreviations

AMSTAR: A MeaSurement Tool to Assess systematic Reviews
GRADE: Grading of Recommendations Assessment, Development and Evaluation
IASP: International Association for the Study of Pain
IMMPACT: Initiative on Methods, Measurement, and Pain Assessment in Clinical Trials
IPT: interdisciplinary pain treatment
PRISMA-P: Preferred Reporting Items for Systematic Review and Meta-Analysis Protocols
PROMIS: Patient-Reported Outcomes Measurement Information System
RCT: randomized controlled trial
VAPAIN: Validation and Application of a core set of patient-relevant outcome domains to assess the effectiveness of multimodal PAIN therapy
